# A Physiotherapeutic Approach to Musicians' Health – Data From 614 Patients From a Physiotherapy Clinic for Musicians (INAP/O)

**DOI:** 10.3389/fpsyg.2021.568684

**Published:** 2021-06-10

**Authors:** Christoff Zalpour, Nikolaus Ballenberger, Florian Avermann

**Affiliations:** ^1^Faculty of Business, Management and Social Science, University of Applied Sciences, Osnabrück, Germany; ^2^Institute for Applied Physiotherapy and Osteopathy, University of Applied Sciences, Osnabrück, Germany

**Keywords:** musicians health, musicians medicine, physiotherapy, manual therapy, osteopathy, performing artists, musicians

## Abstract

Currently, the treatment of musicians is an interprofessional approach. Playing-related health complaints may impact the performance of a musician. In Germany, a medical consulting hour for musicians exists, but those for athletes in sports medicine are not so common. The diagnosing and treatment procedure within the physiotherapy consultation for musicians follows a specific concept-b and requires knowledge of instruments and musician-specific complaints. Based on the consulting hour in a clinic in Osnabrueck, 614 case reports were part of this sample, of which 558 data sets were complete. The focus of the analysis is the instrument and the primary complaint. Also, the type of therapy is characterized, and the amount is calculated. Primary complaints of musicians, in general, are found most frequently in the spine and upper extremity. Musician complaints are different between instruments. Instrumentalists have a significantly higher chance to suffer from a primary complaint in the area of the upper extremity. Furthermore, the groups without an instrument (e.g., singing or dancing) are developing complaints in the anatomical area which they primarily use. Therefore, these types of therapy were used: physiotherapy, manual therapy, and osteopathy with an average of 5.9 treatment units. This study underpinned the importance of musician-specific physiotherapy as a profession to treat musicians. Also, an interdisciplinary approach is necessary to treat all aspects of complaints.

## Introduction

“Musicians Medicine” nowadays is an inter-professional approach of clinicians and artists in helping to recover and maintain musicians' health, including the impact of various disciplines like physicians, psychologists, physical and occupational therapists, amongst others (Sataloff et al., 2010). Playing-related health complaints may impact practice, rehearsals, performance, and even the musical career of music students. They can be considered to result from an impaired interaction of physical, psychological, and psychosocial factors (Fishbein and Middlestadt, 1988; Wesner et al., 1990; Kenny et al., 2004; Rickert et al., 2013; Barbar et al., 2014; Kenny and Ackermann, 2015; Ballenberger et al., 2018).

In this article, the emphasis is put on the impact of physical therapy since epidemiology shows that the highest number of functional disorders in instrumentalists is from (neuro-) musculoskeletal (Fry, 1986; Fishbein and Middlestadt, 1988; Middlestadt and Fisbein, 1989; Brown, 1997; Dawson, 2002; Engquist et al., 2004; Bragge et al., 2005; Fjellman-Wiklund and Chesky, 2006; Abréu-Ramos and Micheo, 2007; Bruno et al., 2008; Hincapié et al., 2008; Ackermann et al., 2012; Gembris and Heye, 2014; Baadjou et al., 2016; Kok et al., 2016), a clinical field where physical therapists are acknowledged as skilled and autonomous practitioners for a long time (Chan et al., 2013a; Chan and Ackermann, 2014).

In a German epidemiological review about work-related strain and disorders in musicians by the occupational physician Irina Böckelmann and the composer Bernard Schneyer (Böckelmann and Schneyer, 2009), both from the Otto-von Guericke University in Magdeburg, in which they prioritized their results by medical subspecialties, the result was quite clear.

The musculoskeletal system was often involved, followed by neurologic problems, dermatologic, hearing loss, psychological, and, finally, many others, including craniofacial problems. Table 1 shows this result concerning possible physical therapy approaches of cure and the strengths and power of certain PT interventions. For each of the three columns, the respective evidence is indicated by the literature (*n* = 79).

**Table 1:** Work-related strain and disorders in musicians and physical therapy approach presented in an earlier version by the first author of this article at the 29th Annual Symposium of the Performing Arts Medicine Association, PAMA in Snowmass/CO 22.07.11 (“A physio clinic for musicians – outcome of the first 200 cases”).

**Disease entity,** prioritized^*^	**LIT**	**Potential power of** ** physio-intervention**	**LIT**	**Examples of physio approaches**	**LIT**
**Musculoskeletal system**					
Repetitive strain/overuse syndromes	Fry, 1986; Fishbein and Middlestadt, 1988; Middlestadt and Fisbein, 1989; Schuppert and Altenmüller, 1999; Liu and Hayden, 2002; Sakai, 2002; Rosety-Rodriguez et al., 2003; Moraes and Papini, 2012; Paarup et al., 2012; Chan and Ackermann, 2014	+++	Ackermann et al., 2002; de Greef et al., 2003; Barton and Feinberg, 2008; Chan et al., 2013a, 2014; López and Martínez, 2013; Chan and Ackermann, 2014; McCrary et al., 2016	Manual therapy, therapeutic exercises, education and advice (e.g., about performance posture, practice habits, possible injury risks etc.), music performance biomechanics feedback, ergonomic considerations	Ackermann et al., 2002; de Greef et al., 2003; Shafer-Crane, 2006; Rabuffetti et al., 2007; Barton and Feinberg, 2008; Chan et al., 2013b; López and Martínez, 2013; Chan and Ackermann, 2014; McCrary et al., 2016
Mobility dysfunction	Fishbein and Middlestadt, 1988; Middlestadt and Fisbein, 1989; Larsson et al., 1993; Gannon and Bird, 1999; Schuppert and Altenmüller, 1999; Brandfonbrener, 2002; Liu and Hayden, 2002; Sakai, 2002; Day et al., 2011; Driscoll and Ackermann, 2012; Paarup et al., 2012; Artigues-Cano, 2014; Chan and Ackermann, 2014; Skwiot et al., 2019	+++	de Greef et al., 2003; Barton and Feinberg, 2008; Bathen et al., 2013; Chan et al., 2013a, 2014; Scheper et al., 2013; Chan and Ackermann, 2014; Engelbert et al., 2017	Manual therapy, therapeutic exercises, education and advice (e.g., about performance posture, practice habits, possible injury risks etc.) music performance biomechanics feedback, ergonomic considerations	Russek, 2000; de Greef et al., 2003; Barton and Feinberg, 2008; Bathen et al., 2013; Chan et al., 2013b; Scheper et al., 2013; Artigues-Cano, 2014; Chan and Ackermann, 2014; Engelbert et al., 2017
**Nervous system**					
Nerve dysfunction	Fishbein and Middlestadt, 1988; Hoppmann and Patrone, 1989; Lederman, 1989; Claus and Schaller, 1993; Schuppert and Altenmüller, 1999; Liu and Hayden, 2002; Kennedy et al., 2006; Paarup et al., 2012; Wilson et al., 2014; Demaree et al., 2017	+++	Claus and Schaller, 1993; Oskouei et al., 2014; Wilson et al., 2014	Neuro mobilization/neural dynamics techniques, Manual therapy/passive mobilization, education and advice (regarding playing schedules, practice habits, instrument modification, activity modification), ergonomic considerations, splinting, posture correction, active exercises	Hoppmann and Patrone, 1989; Claus and Schaller, 1993; Oskouei et al., 2014; Wilson et al., 2014
Focal dystonia	Lederman, 1991; Schuppert and Altenmüller, 1999; Bowie et al., 2000; Lahme et al., 2000; Altenmüller and Jabusch, 2002, 2010; Liu and Hayden, 2002; Schuele and Ledermann, 2004; Aránguiz et al., 2011; Furuya and Altenmüller, 2015	++	Berque et al., 2013; Enke and Poskey, 2018	Motor control retraining	Schuele and Ledermann, 2004; Aránguiz et al., 2011; Berque et al., 2013; Enke and Poskey, 2018
**Dermatology** e.g., contact eczema	Buckley and Rogers, 1995; Moreno et al., 1997; Adams, 2000; Liu and Hayden, 2002	0			
**Hearing disability**	Marquard and Schäcke, 1998; Schuppert and Altenmüller, 1999; Kähäri et al., 2001; Lockwood et al., 2001; Strasser, 2004; Olson et al., 2016	+	Hülse and Hölzl, 2004; Arif et al., 2017	Relaxation (tinnitus), enhancing awareness for prevention strategies	Lockwood et al., 2001; Hülse and Hölzl, 2004; Olson et al., 2016; Arif et al., 2017
**Psycho and Psycho-somatic Disorders**	Fishbein and Middlestadt, 1988; Möller, 1990, 1997; Schuppert and Altenmüller, 1999; Fjellman-Wiklund et al., 2003; Kenny et al., 2004, 2016; Wristen and Fountain, 2013		Su et al., 2010	Relaxation, body-awareness, breathing exercises	Su et al., 2010
**Others**					
e.g., cranioman-dibular dysfunction /facial pain	Hirsch and McCall, 1982; Schuppert and Altenmüller, 1999; Liu and Hayden, 2002; Steinmetz et al., 2003; Rodríguez Lozano et al., 2010, 2011; Heikkilä et al., 2012; van Selms et al., 2019	+++	Calixtre et al., 2015; Armijo-Olivo et al., 2016; Martins et al., 2016; Shimada et al., 2019	Manual Therapy (special treatment incl. myofascial release, cervical spine mobilization), therapeutic exercises (incl. mobilization exercise, strengthening, coordination, and postural exercise)	Calixtre et al., 2015; Armijo-Olivo et al., 2016; Martins et al., 2016; Shimada et al., 2019

* *according to Böckelmann and Schneyer (2009)*.

### Provision of Musician Specific Care in Germany

Medical consulting hours for musicians are existent in Germany though happening on a very low scale compared with those for athletes in sports medicine (Spahn et al., 2010). Approximately almost a dozen Music-Universities physicians are engaged in either full-time occupancy or as consultants in diagnosing and treating musicians. In many cases, those are run by Professors for Music Physiology and Musicians Medicine who are double-skilled: with a degree in music and another in a sub-discipline of the medical field, e.g., neurology, (hand-)surgery, occupational medicine or psychiatry amongst others. At the *University of Applied Sciences in Osnabruck*, there is no post like this, but the unique coincidence with a strong department of music (all genres) and physiotherapy. In 2007 advantage was made out of this situation and establishing a physio clinic (INAP/O) within the university of applied sciences. Shortly after the place's opening (located directly on the first floor in the School of Music (Institut für Musik), a specific need for physiotherapeutic treatment of musicians was obvious. Intrigued by the strong evidence of physical therapy underpinned in Table 1, a specific concept for diagnosing and treating musicians was developed and specialized during the following years. Caregivers are, without exception, trained physical therapists in the field of musicians' physiotherapy that were also musicians by themselves. Using different refinancing models (e.g., study fees, grant-support, and special engagement of the university itself), the service is free of charge for music students of the institute of music at the university. Other musicians who use the clinic are either involved in ongoing research projects (and then receiving examination and treatment for free) or having their health insurances (public or private) paying for the service. The vast amount of clients are music students, though.

Since the profound knowledge of instrument-specific complaints is crucial for specially tailored, individualized, and effective treatment, the authors of this article were questioning themselves whether instrument- and body region-specific complaints can be detected among musicians attending physiotherapeutic consulting hour. Therefore, data comparison was based on more than 600 case reports.

What physiotherapy approaches in terms of different concepts would be used in a clinical setting for diagnosing and treating musicians concerning the physio approaches mentioned in Table 1.

## Methods

### The Physiotherapeutic Consulting Hours

The diagnosing and treatment procedure within the physiotherapeutic consulting hours for musicians follows a specific concept: as usual in Germany, one “unit” of physiotherapy contains 25 min. Therefore, the first visit is always scheduled for 50 min. Musicians usually bring their instruments to the appointment (except pianists, instrument is provided by the INAP/O). The physical examination is carried out with the instrument in both sitting and standing positions. If necessary, musicians get six units of PT per semester for free (12/a). If there is a need for more, they will receive treatment based on prescriptions by resident doctors (which enables payment for PT service by public/private insurances).

Each musician who wishes to receive physiotherapy or osteopathy specialized for musicians must sign the informed consent paper. With the signature, the patient agrees to the terms of the musician physiotherapy consulting hour, including the anonymous data collection for the study in a specialized findings sheet.

Following parameters are collected with the findings sheet:

- age at the first appointment
- main instrument and, if necessary second, or any further
- also, other instruments if they play it as a hobby
- history of the main instrument
  ° differences in style of playing
  ° start to learn the instrument professionally
  ° start of playing
  ° playing time in the history
  ° different teachers over time
- genre of playing
- pain during playing
- subjective description of the primary complaint and its location

Also, the therapist decides the type of treatment (e.g., physiotherapy, osteopathy, manual therapy) and how many appointments are needed.

### Data Collection and Processing

All these parameters are collected by the therapists directly and safely stored just for scientific use. To digitize the data, a self-made Javascript tool was used, storing the data in a Microsoft Excel format. For analyzing Microsoft Excel 365 and SPSS, Version 25 by IBM was used.

Before the statistical analysis, the data were split into six groups to perform group comparisons. The superior instrument groups (including one for **s**inging and one for dancing) (IGSD) are the following:

- piano
- wind (clarinet, saxophone, flute, trumpet, bassoon, trombone, recorder, French horn)
- string (guitar, viola, cello, violin, double bass, bass, electric guitar, electric bass, harp)
- percussion (drums, marimba)
- singing and
- musical (includes dancing in particular besides singing)

The four superior complaint groups are the following:

- head + face + jaw
- upper extremity
- spine and
- lower extremity

### Data Analysis

The statistical analysis is divided into four parts. First, the significance level α is set to 0.05.

The null hypothesis is rejected if *p* < 0.05.

To check if the baseline characteristics like sex and age differ significantly between music disciplines, Chi^2^-test, and analysis of variance were used.

The number of body region-specific complaints and their distribution depending on IGSD were analyzed descriptively and presented as frequencies. To ensure the possibility to compare the IGSD, the frequencies are weighted for the number of patients in each group.

As a second part, a binary logistic regression has been performed to compare the chances of having body-specific complaints according to IGSD. Odds ratios, including confidence intervals, were calculated between pairs of IGSD. Odds ratios in which the one value is not intersecting the interval were considered significant group comparisons. Age and sex were included in the model as covariates to adjust potential confounding.

The third part comprised a multinomial logistic regression to calculate the predicted probabilities of having a body-specific complaint depending on IGSD adjusted for age and sex in the model. Based on that, colored body charts were designed depending on the respective predicted probability (see **Figure 2**).

## Results

All the data were collected between 2008 and 2018. In this time, 614 different musicians were part of the musician-specific physiotherapy consulting hour. Due to missing complete datasets of some patients, more than 550 patients could finally be considered for the analysis. The sex distribution was almost equal (50.9% female and 49.1% male). The mean age and standard deviation of the participants were 23.99 ± 4.14 years. Between the IGSD, the mean age ranges from 22.7 to 24.4 years (Table 2). There is no significant difference in age (*p* = 0.236); however, sex is significantly different between groups (*p* ≤ 0.001).

**Table 2:** Age and sex depending on IGSD.

**IGSD**	**Distribution**	**Age**	**Sex (percentage)**
	**(percentage)**	**(mean + SD)**	
Wind instruments	11.3 (*n* = 63)	23.9 ± 4.25	m: 47.62; f: 52.38
String instruments	26.3 (*n* = 147)	24.37 ± 4.8	m: 64.63; f: 35.37
Percussion instruments	13.3 (*n* = 74)	23.76 ± 3.47	m: 93.24; f: 6.76
Singing	27.4 (*n* = 153)	23.97 ± 3.75	m: 16.99; f: 83.01
Musical	8.2 (*n* = 46)	22.67 ± 1.91	m: 36.96; f: 63.04
Piano	13.4 (*n* = 75)	24.39 ± 4.88	m: 49.33; f: 50.67

On average, musicians received 5.9 treatment units. A total of 2,984 treatments were given during this period. Of these, 23.3% were physiotherapy, 58.6% manual therapy, and 18% osteopathy. The types of therapy described in Table 1 are used daily by the therapists to ensure the best possible care for the musicians. Despite every treatment, the matter of therapy (physiotherapy, osteopathy, manual therapy) is education and advice about performance posture and practice habits. In addition, every musician will be analyzed during performing with their instrument if possible. The treatment techniques of neuromobilization, neural dynamics techniques, or cervical mobilization are central to manual therapy and osteopathy. Osteopathy differs from manual therapy in possible treatment approaches. Osteopathy sees a connection between musculoskeletal complaints and the visceral system. Techniques like strengthening and exercising are considered to belong to basic physiotherapy.

The primary complaint of each patient was classified into four body regions. The most primary complaint is the spine area, with 57.7%. The lowest occurrence is in the lower extremity area (7.9%). These complaints are differently distributed between the IGSD.

In the head + face + jaw area, the singers are the most frequent with 36.4%, and the lowest score is 6.2% for piano.

In the area of the upper extremity, the string instrument players (25.7%) appear most often, and the lowest percentage has musical with 5.7%.

The distribution of the IGSD in the spinal area is between 14.5 and 18.4%.

**Figure 1:**
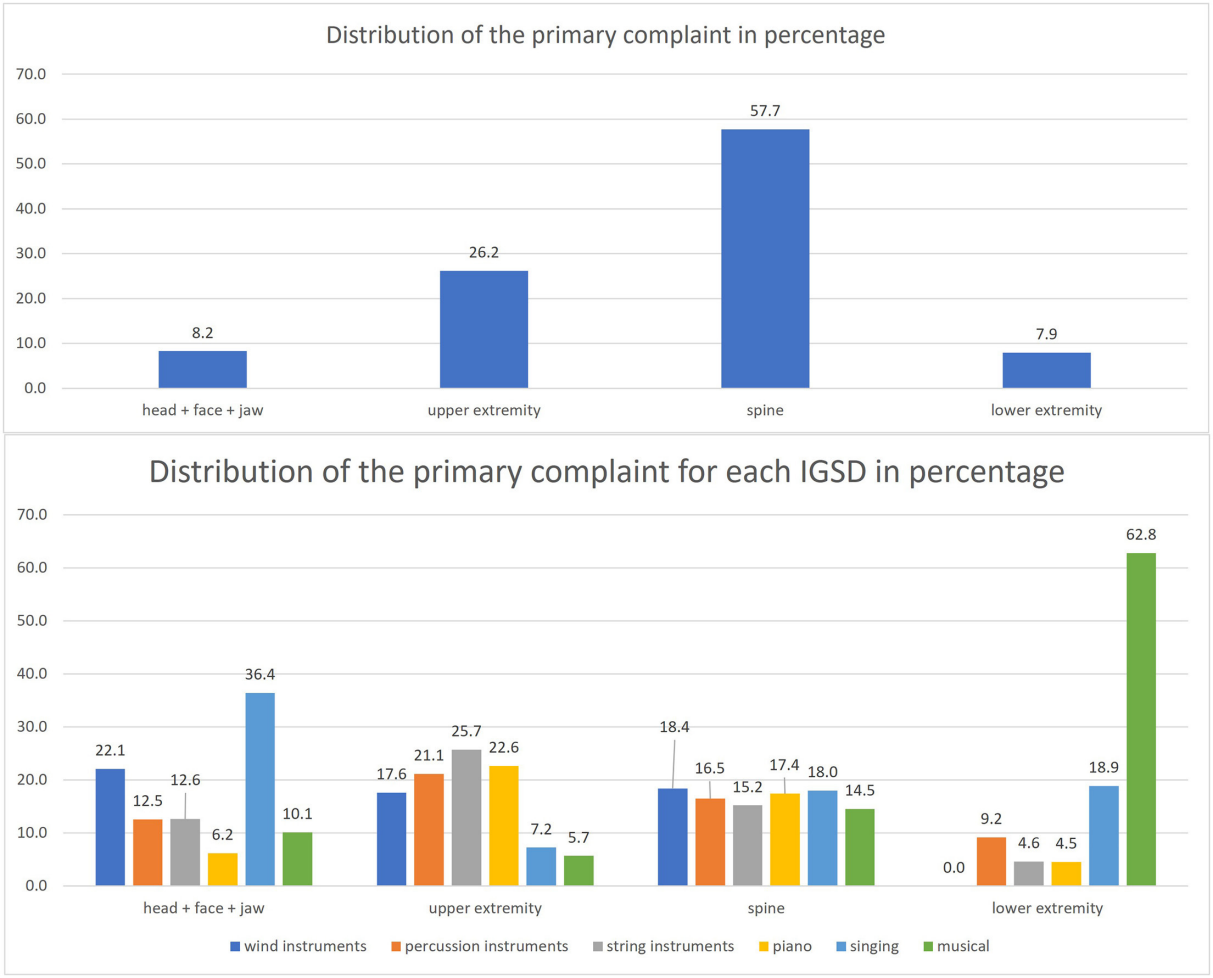
Distribution of the primary complaint.

The last group of the primary complaint is the lower extremity. Here the musical artists are the most frequent with 62.8%, and the rest ranges from 0 to 18.9%. The following Figure 1 shows the distribution of the primary complaint in the sample and is split into the IGSD.

In Table 3, the odds ratios for the comparison of body-specific complaints between pairs of instrument groups based on logistic regression adjusted for age and sex. The odds ratios are to be interpreted as follows: Odds ratios greater than one are associated with a higher chance of experiencing complaints compared between pairs of instrument groups. Accordingly, odds ratios less than one is associated with a decreased chance of experiencing complaints. For example, the chance for a singer to gain a primary complaint in the head + face + jaw area is 6.8 times higher than the chance for a piano player (Table 3A).

**Table 3:** Odds ratios of experiencing complaints compared between IGSD based on logistic regression.

	**Piano**	**Wind**	**String**	**Singing**	**Percussion**	**Musical**
**A** **=** **HEAD** **+** **FACE** **+** **JAW**						
Piano		0.26 (0.05;1.34)	0.48 (0.1;2.3)	**0.15 (0.03;0.64)**	0.48 (0.09;2.7)	0.6 (0.08;4.43)
Wind	3.85 (0.75;19.61)		1.83 (0.61;5.51)	0.57 (0.22;1.46)	1.84 (0.5;6.86)	2.316 (0.45;12.03)
String	2.1 (0.44;10.1)	0.55 (0.18;1.65)		**0.31 (0.13;0.71)**	1.01 (0.29;3.46)	1.27 (0.26;6.19)
Singing	**6.8 (1.56;29.41)**	1.77 (0.69;4.57)	**3.24 (1.4;7.46)**		**3.26 (1.09;9.76)**	4.09 (0.93;18.03)
Percussion	2.09 (0.37;11.77)	0.54 (0.15;2.02)	0.99 (0.29;3.41)	**0.31 (0.1;0.92)**		1.257 (0.22;7.15)
Musical	1.66 (0.23;12.12)	0.43 (0.08;2.24)	0.79 (0.16;3.86)	0.24 (0.06;1.08)	0.8 (0.14;4.53)	
**B** **=** **UPPER EXTREMITY**						
Piano		1.43 (0.68;3.01)	0.85 (0.47;1.53)	**3.48 (1.67;7.23)**	1.51 (0.7;3.28)	**5.26 (1.68;16.42)**
Wind	0.7 (0.33;1.47)		0.59 (0.31;0.45)	**2.5 (1.13;5.54)**	1.01 (0.43;2.35)	**3.69 (1.14;11.94)**
String	1.17 (0.65;2.11)	1.7 (2.25;3.28)		**4.84 (2.48;9.43)**	1.41 (0.76;2.61)	**6.63 (2.23;19.75)**
Singing	**0.29 (0.14;0.6)**	**0.4 (0.18;0.89)**	**0.21 (0.11;0.4)**		0.38 (0.14;1.07)	1.46 (0.45;4.73)
Percussion	0.66 (0.31;1.44)	0.99 (0.43;2.31)	0.71 (0.38;1.32)	2.62 (0.94;7.3)		3.65 (0.97;13.77)
Musical	**0.19 (0.06;0.59)**	**0.27 (0.08;0.88)**	**0.15 (0.05;0.45)**	0.68 (0.21;2.21)	0.27 (0.07;1.03)	
**C** **=** **LOWER EXTREMITY**						
Piano		n.a.	0.98 (0.18;5.47)	**0.22 (0.05;0.98)**	0.48 (0.09;2.7)	**0.05 (0.01;0.22)**
Wind	n.a.		n.a.	n.a.	n.a.	n.a.
String	1.02 (0.18;5.71)	n.a.		**0.22 (0.07;0.68)**	0.49 (0.12;2.02)	**0.05 (0.02;0.15)**
Singing	**4.57 (1.03;20.41)**	n.a.	**4.46 (1.47;13.7)**		2.19 (0.71;6.75)	**0.21 (0.1;0.47)**
Percussion	2.09 (0.37;11.77)	n.a.	2.04 (0.5;8.4)	0.46 (0.15;1.4)		**0.1 (0.03;0.32)**
Musical	**21.28 (4.65;100)**	n.a.	**20.83 (6.58;66.67)**	**4.7 (2.15;10.31)**	**10.31 (3.18;33.33)**	

*It reads like this: e.g., The chance of a lower extremity complaint is 21.28 times higher among musical artists when compared with piano. Accordingly, the chance of a lower extremity complaint about a piano player is 0.05 the chance for a musical artist. n.a., not applicable*.

*The bold values are significant results. p < 0.05*.

The statistical model of the logistic regression for the primary complaint “head + face + jaw” is highly significant with a *p*-value of 0.005 (Table 3A). Thus, the chance of having a head + face + jaw complaint is 3.24 times higher among singers when compared with string instrument players.

The differences in the primary complaint “upper extremity” are highly significant (*p* ≤ 0.001) (Table 3B). For example, the chance of having an upper extremity complaint is 4.8 times higher among string instrument players when compared with singers and 6.6 times higher compared with musical artists.

The statistical model for the “spine” as the primary complaint showed no significant differences between the various IGSD (*p* = 0.455). Therefore, the odds ratios for the spine area are not demonstrated.

The statistical model for the “lower extremity” group is highly significant (*p* ≤ 0.001) (Table 3C). There are no results for the wind instrument group because the count of patients within this group was too small. As shown in Table 3, the musical artists have the highest chance for a complaint around the lower extremity compared with the other instrument groups.

**Figure 2:**
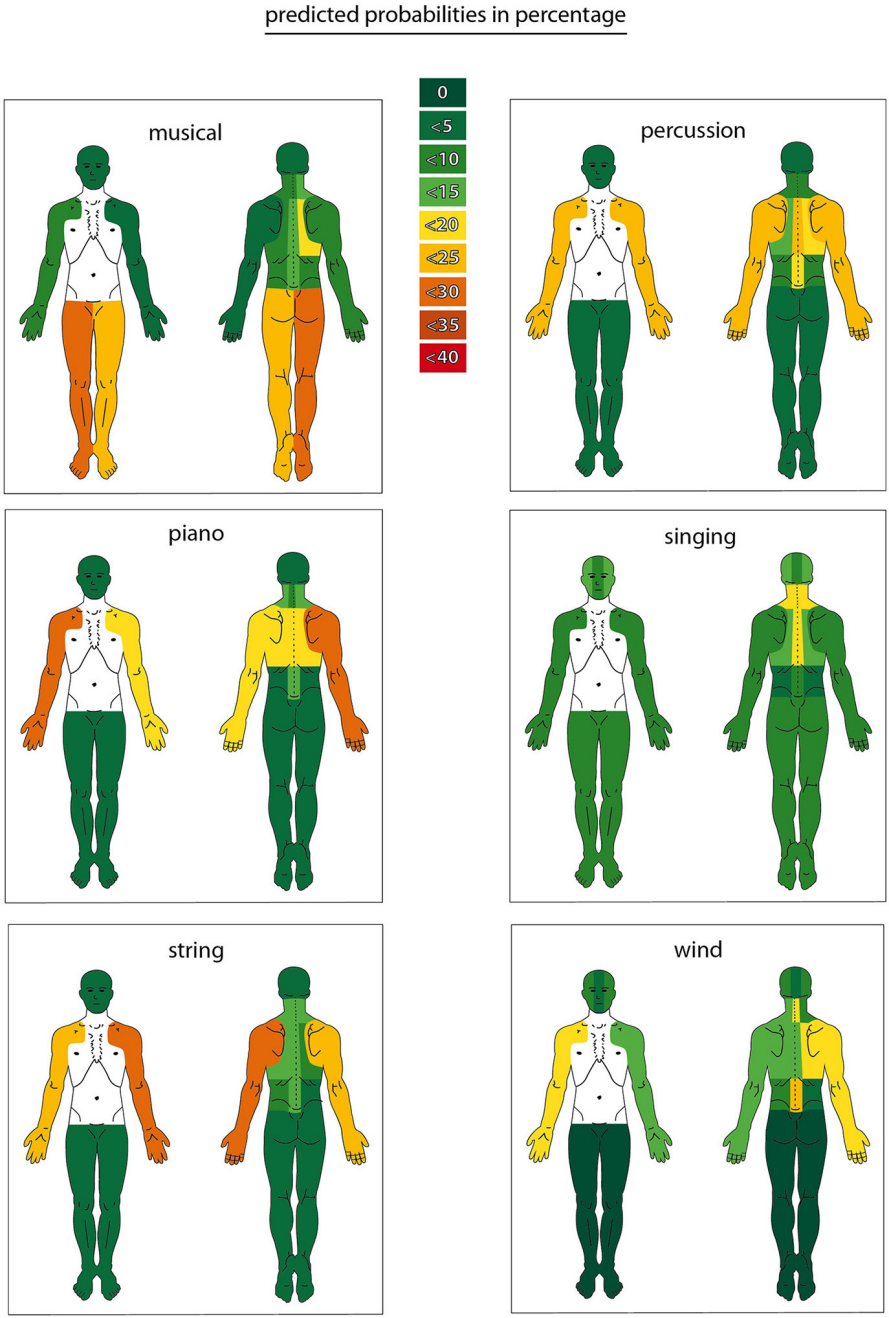
Predicted probabilities for primary complaints in every IGSD.

In the third part of the analysis, predicted probabilities for a complaint were calculated, taking into account side differences as shown in Figure 2. The spine, in general, has the highest percentage of every area. The range is between 29 and 51% for the cervical spine. The thoracic spine is between 33 and 53%, and the lumbar spine between 16 and 31%. The upper extremity ranges between 12 and 28% for musicians with an instrument (piano, wind, string, percussion). The highest predicted probabilities for the lower extremity occur among the musical artists (24–26%). The area head, face, jaw ranges between 1 and 12%. The highest predicted probabilities are found among the singing group. The highest values for a piano player are found around the upper extremity (right: 28.5%, left: 18.5%). In the wind, instrument group exists a side difference in the upper extremity (right: 15.9%, left: 12.4%). The highest values for the string instrument group are found in the upper extremity area (right: 21.2%, left: 28.2%). In the singing group, the cervical and thoracic spine reaches high values up to 18% and the head + face + jaw area up to 12%. In percussion instruments, the predicted probabilities for the right upper extremity are 21.7% and the left 22.8%. The musical artists show high values in the lower extremity area (right: 24%, left: 26%).

## Discussion

The study results show how primary complaints differ from the type of instrument, except spinal complaints closely distributed between groups. The most common area is the spine, especially the cervical spine (up to 51%). This result is already supported by the literature (Steinmetz et al., 2015; Cruder et al., 2018, 2021). Steinmetz et al. showed in 2018 in a large survey that the most frequent complaint area is the cervical spine (Steinmetz et al., 2015). In comparison, the results of Steinmetz et al. show a lot higher frequencies in the different areas. This difference can be explained by the fact that they have also asked about the past and not just asked about the current situation.

The upper extremity complaints are more frequent in the groups than singers and dancers (musical artists). The odds ratios support the findings. For example, a viola or violin player must do very precise and repetitive movements in an asymmetric posture where they hold their instrument between the chin and the shoulder. This could contribute to developing pain in this area (Rensing et al., 2018). Furthermore, the different loads for both upper extremities can explain the contrast between right and left.

The singing group has its most frequent complaint in the head + face + jaw area. The load in this area while performing is different to, for example, string instruments. The odds ratios reflect this. Pairwise comparison is only significant for those instruments with lower loads in this area while playing (Table 3A).

The lower extremity is affected the most in the musical artists' group. The majority are students. A huge part of the degree program musical at the university of applied sciences is ballet, jazz, and tap dance. Therefore, the load is more comparable to a dancer. Also, in a retrospective study about musculoskeletal injuries in young ballet dancers by Leanderson et al., the result shows a higher occurrence for injuries in the lower extremity (Leanderson et al., 2011). The odds ratios support this outcome. Therefore, the chance for musical artists for a primary complaint in this area is a lot higher.

In Kok et al., the highest occurrence of musicians' complaints is in the neck and shoulder area (Kok et al., 2016). Compared with this study, the spine was the most frequent complaint, followed by the upper extremity, including the shoulder area. A distinction between playing-related complaints vs. other complaints was not possible with our data. So, the reported results cannot generate a direct association between the complaints and the instrument. But the current literature supports this association without pointing to side differences (Kok et al., 2016). In this study, the predicted probabilities are showing side differences. Ackermann et al. have already shown in 2012 that string players reported pain on the left upper extremity more often (Ackermann et al., 2012). Our findings support those results. Playing a string instrument comes with a significant difference between the right and left arm (Rensing et al., 2018). If the different tasks are the reason for the high numbers of the predicted probabilities, future research needs to be addressed.

Table 1 shows the huge variety of playing-related complaints and how different physio approaches can address them. This supports the idea of establishing specific physiotherapy consulting hours. One important part of the treatment is proper education (e.g., on ergonomic posture) and advice (e.g., on the importance of breaks), which was proofed to be effective (Chan et al., 2013a). In our study, this was integrated into the therapy approach (present in all treatment groups, physiotherapy, manual therapy, and osteopathy), but we didn't measure its effectiveness. Through clinical experience, we would strongly follow Chan et al. in valuing the necessity of it. Hoppmann and Patrone already showed in their review from 1989 that commonly musculoskeletal injuries in musicians are, e.g., musculotendinous overuse or nerve entrapment (Hoppmann and Patrone, 1989). In addition, Oskouei et al. showed that physiotherapy alone and physiotherapy and neuro mobilization maneuver are effective in treating carpal tunnel syndrome (Oskouei et al., 2014). As mentioned above, neuro mobilization is a part of manual therapy and osteopathy. In a study by Lederman from 2006, he describes an instrument adaptation to reduce the upper extremity symptoms of one patient (Lederman, 2006). Those examples underline the importance of a specialized approach for the treatment of musicians. Therapists need to be aware of the possibility that the instrument can be a contributing factor to their patients' symptoms.

One strength of this study is the amount of data (*n* = 558). These data were collected in one study center (the INAP/O), so the transferability to the practice's patients is possible and can help ensure better treatment for musicians. In addition, this result can support non-specialized physiotherapists to be aware of the possibility of a connection between a patients' complaint and an instrument. Furthermore, the statements of this study will be clearer as the amount of data will increase over time. This will affect collecting data on rare instruments as well.

One weakness of the study is the missing data. Not all designated data sets were complete, and so limiting the analysis. The missing data of the playing time is a flaw as it can impact the development of a primary complaint (Robitaille et al., 2018). Due to the connection described above (mostly music students from on university), the sample is highly selective, and the transferability to other groups is restricted, limiting the generalizability.

The research shows that the primary complaint does not have to be in the musculoskeletal system. Psychologic aspects like performance anxiety, depression, or stress can be factors for musicians (Kenny et al., 2004; Kenny and Ackermann, 2015; Ballenberger et al., 2018). Hence a more interdisciplinary approach to treat the complaints of musicians must be considered. In the future data collection procedure, musician-specific psychological questionnaires will be implemented to record stress and performance anxiety. The interference between musculoskeletal complaints and psychological factors should be searched for in the future, ensuring a more individualized approach.

The results of the study present the variability of primary complaints about musicians and possible covariates. Physiotherapy can have a possible positive influence and should be considered an independent profession in an interdisciplinary approach to the complaint management of musicians. On average, each musician received 5.9 treatment units. In the next step, the research at the physiotherapy consultation at the INAP/O will focus on the effectiveness after a different number of treatment units. In addition, possible questionnaires will be implemented to detect potential cofounders, like psychological factors as mentioned above. Especially the lack of information about the effectiveness of therapy should be considered as one of the main topics for further research. Therefore, longitudinal studies are required to fill this gap in research.

## Data Availability Statement

The raw data supporting the conclusions of this article will be made available by the authors, without undue reservation.

## Ethics Statement

The studies involving human participants were reviewed and approved by the ethics committee of Osnabrück University of Applied Sciences. The participants provided their written informed consent to participate in the study.

## Author Contributions

All authors listed have made a substantial, direct and intellectual contribution to the work, and approved it for publication.

## Conflict of Interest

The authors declare that the research was conducted in the absence of any commercial or financial relationships that could be construed as a potential conflict of interest.
